# A geospatial approach to identify patterns of antibiotic susceptibility at a neighborhood level in Wisconsin, United States

**DOI:** 10.1038/s41598-023-33895-5

**Published:** 2023-05-02

**Authors:** Laurel Legenza, Kyle McNair, Song Gao, James P. Lacy, Brooke J. Olson, Thomas R. Fritsche, Lucas T. Schulz, Samantha LaMuro, Frances Spray-Larson, Tahmeena Siddiqui, Warren E. Rose

**Affiliations:** 1 School of Pharmacy, University of Wisconsin–Madison, 777 Highland Avenue, Madison, WI 53705 USA; 2 State Cartographer’s Office, Department of Geography, University of Wisconsin–Madison, Madison, WI USA; 3 Department of Geography, University of Wisconsin–Madison, Madison, WI USA; 4 Marshfield Clinic Health System, Marshfield, WI USA; 5 UW Health, Madison, WI USA; 6 Fort HealthCare, Fort Atkinson, WI USA

**Keywords:** Infectious diseases, Bacterial infection, Clinical microbiology

## Abstract

The global threat of antimicrobial resistance (AMR) varies regionally. This study explores whether geospatial analysis and data visualization methods detect both clinically and statistically significant variations in antibiotic susceptibility rates at a neighborhood level. This observational multicenter geospatial study collected 10 years of patient-level antibiotic susceptibility data and patient addresses from three regionally distinct Wisconsin health systems (UW Health, Fort HealthCare, Marshfield Clinic Health System [MCHS]). We included the initial *Escherichia coli* isolate per patient per year per sample source with a patient address in Wisconsin (N = 100,176). Isolates from U.S. Census Block Groups with less than 30 isolates were excluded (n = 13,709), resulting in 86,467 *E. coli* isolates. The primary study outcomes were the results of Moran’s I spatial autocorrelation analyses to quantify antibiotic susceptibility as spatially dispersed, randomly distributed, or clustered by a range of − 1 to + 1, and the detection of statistically significant local hot (high susceptibility) and cold spots (low susceptibility) for variations in antibiotic susceptibility by U.S. Census Block Group. UW Health isolates collected represented greater isolate geographic density (n = 36,279 *E. coli*, 389 = blocks, 2009–2018), compared to Fort HealthCare (n = 5110 isolates, 48 = blocks, 2012–2018) and MCHS (45,078 isolates, 480 blocks, 2009–2018). Choropleth maps enabled a spatial AMR data visualization. A positive spatially-clustered pattern was identified from the UW Health data for ciprofloxacin (Moran’s I = 0.096, p = 0.005) and trimethoprim/sulfamethoxazole susceptibility (Moran’s I = 0.180, p < 0.001). Fort HealthCare and MCHS distributions were likely random. At the local level, we identified hot and cold spots at all three health systems (90%, 95%, and 99% CIs). AMR spatial clustering was observed in urban areas but not rural areas. Unique identification of AMR hot spots at the Block Group level provides a foundation for future analyses and hypotheses. Clinically meaningful differences in AMR could inform clinical decision support tools and warrants further investigation for informing therapy options.

## Introduction

Antimicrobial Resistance (AMR) is one of the greatest health challenges of our time^1,2^. AMR not only threatens our ability to treat infections for all populations, but AMR also jeopardizes our ability to survive routine surgeries and immunocompromising conditions. The Centers of Disease Control and Prevention (CDC) estimate that over two million people are affected by AMR infections each year, and more than 35,000 people die from AMR infections annually in the United States^1,3^. To mitigate this threat, the CDC and the Infectious Disease Society of America recommend improved surveillance methods and appropriate antibiotic use^4^. A gap remains in the availability of AMR surveillance data at local levels and in clinical practice^5^.

Importantly, resistance rates can vary across a country or even within the same state, as reported by the CDC nationally and locally in Wisconsin^6–8^. Globally, differences in antibiotic susceptibility can be observed and linked with local antibiotic use in healthcare and agriculture^9–11^. Our prior research identified geospatial patterns across Wisconsin when analyzing AMR data at the health system level^12^. Statistical modeling of AMR data nationally also shows variations regionally, by groups of U.S. states^13^. A gap remains in AMR data at smaller units of geography such as at city or neighborhood levels, especially in the United States. The few infectious diseases-related geospatial studies at neighborhood levels were performed primarily in Brazil and Ireland and were limited to the specific isolates or data collected for each study^14–17^.

Regional differences in antibiotic susceptibility may be related to socio-economic factors, including access to healthcare, food, and antibiotic use. Studies in England, including one with the Index of Multiple Deprivation (IMD), found overall antibiotic prescribing was higher in areas with greater deprivation, but broad spectrum antibiotic prescribing was higher in affluent areas, and found significant variations in prescribing regionally^18,19^. Regional income differences in Germany were associated with differences in pediatric antibiotic prescribing^20^. These regional differences in medication access and healthcare exposure may impact antimicrobial susceptibility and the occurrence of multidrug resistant infections. For example, a case control study of pediatric patients presenting to emergency departments found distinct geographic clustering of community-onset methicillin-resistant *Staphylococcus aureus* cases compared to non-infectious controls, along with differences associated with race, age, and type of health insurance^21^.

In this study, we use spatially-enabled tools to map and analyze antimicrobial susceptibility from health systems by U.S. Census Block Group (hereafter, Block Group). We describe methods for identifying patterns of AMR across urban and rural areas at this granular level. We hypothesize that AMR spatial distribution is not random in the geographic areas served by Wisconsin health systems. This unique identification of AMR hot-spots at the Block Group level provides a foundation for future analyses and hypotheses.

## Results

UW Health isolates meeting inclusion criteria represented more urban areas, more Block Groups, and greater isolate geographic density (n = 36,279 isolates, 389 blocks, 2009–2018) compared to Fort HealthCare (n = 5110 isolates, 48 blocks, 2012–2018) and MCHS (n = 45,078 isolates, 480 blocks, 2009–2018). Block Groups with less than 30 isolates were excluded. Table 1 shows the total number of isolates collected with the corresponding number of Block Groups for each health system and the total separated by excluded and included Block Groups.

**Table 1:** *Escherichia coli* isolates collected, excluded, and included with antibiotic susceptibility results by health system.

	Marshfield clinic health system	UW health	Fort healthcare
Total isolates collected, no.	49,482	44,629	6065
Total census Block Groups represented, no.	1280	2302	415
Excluded			
Excluded isolates, no. (%)	4404 (9)	8350 (19)	955 (16)
Excluded census Block Groups (< 30 isolates), no. (%)	800 (63)	1913 (83)	367 (88)
Included			
Included isolates, no. (%)	45,078 (91)	36,279 (81)	5110 (84)
Included census Block Groups (≥ 30 isolates), no. (%)	480 (38)	389 (17)	48 (12)
Antibiotic susceptibility			
Ciprofloxacin susceptibility mean, % (census block group range, SD)	90 (63–100, 6)	88 (62–100, 6)	84 (67–100, 8)
Sulfamethoxazole/trimethoprim susceptibility, % (census block group range, SD)	87 (69–100, 5)	82 (55–100, 6)	82 (68–94, 5)

**Figure 1:**
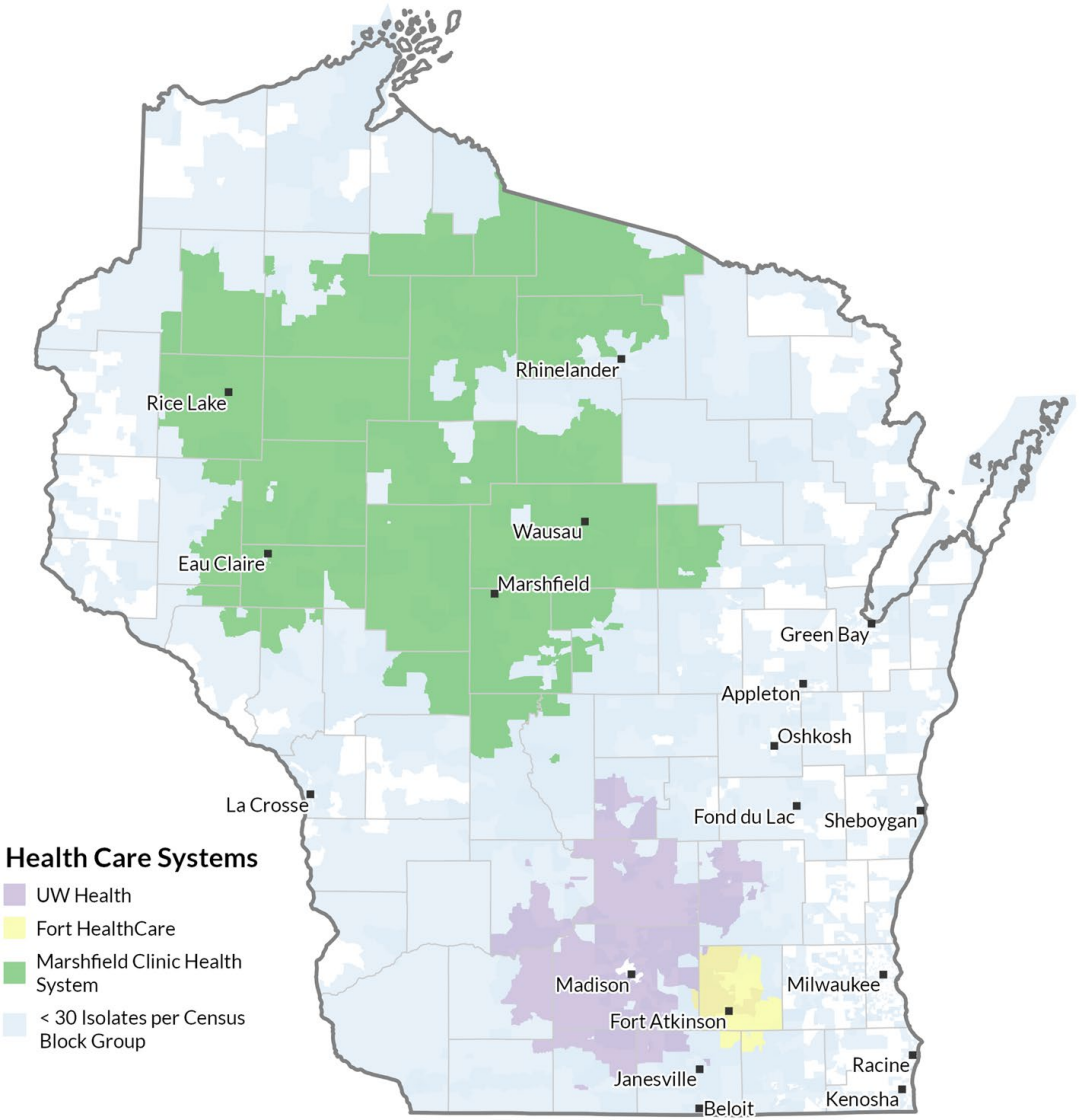
Wisconsin study areas included and excluded in analysis*. Isolate counts less than and greater than 30 isolates by U.S. Census Block Groups for each health system are shown. Block Groups with at least 30 isolates were included in the analysis. Figure created with ArcGIS Pro software (Version 2.7; ESRI, Redlands, California, https://www.esri.com/en-us/arcgis/products/arcgis-pro/overview). *This geographic data presentation is considered ‘de-identified’ by ‘Expert determination’ under the HIPAA Privacy rule.

The Block Groups included and excluded for each health system are shown within a map of Wisconsin in Fig. 1. Most sample results represented areas near the health systems’ primary locations: UW Health represented the greater Madison area; Fort HealthCare data was from the Fort Atkinson metropolitan area; MCHS data covered the largest geographic area across north-central Wisconsin. Figure 1 shows how most isolates included were from concentrated areas within and around cities.

Many Block Groups covering both rural and urban areas from all three health systems were excluded because they did not have enough isolates (Table 1). Some counties are completely blue counties in Fig. 1, meaning that at least one isolate was collected across the three health systems from every Block Group in the county, yet the county was still excluded because the Block Groups had less than 30 isolates. The included and excluded areas align with the primary service area of each health system. For example, both rural and urban areas throughout the eastern side of Wisconsin were excluded (Fig. 1). The predominate health systems in eastern Wisconsin were not included in the study”.

### Susceptibility data visualization

**Figure 2:**
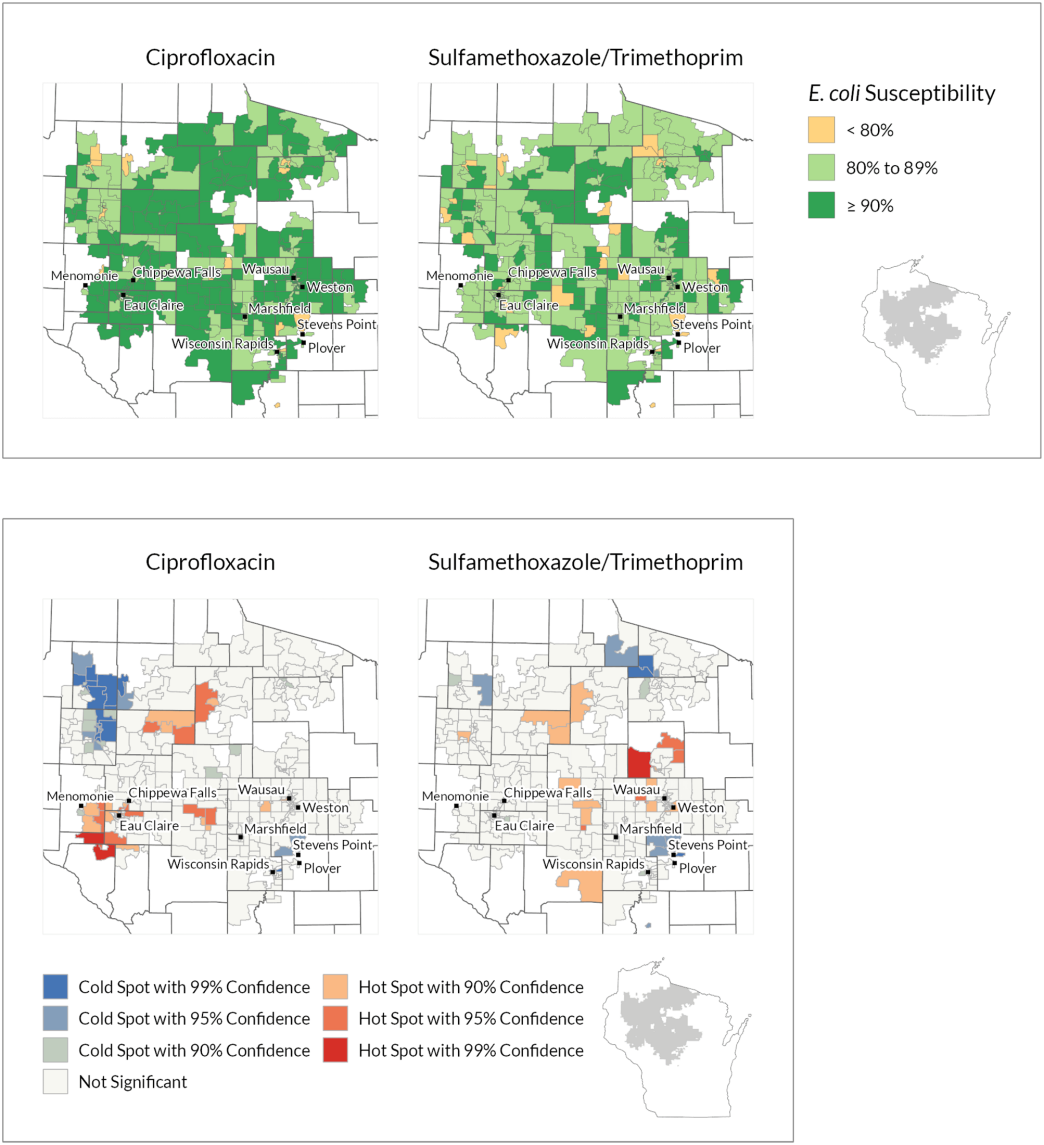
Marshfield Clinic Health System ciprofloxacin and sulfamethoxazole/trimethoprim susceptibility choropleth (noncontiguous areas included)^a^ and hot spot analysis results (Contiguous edge only)^b^*. ^a^Colored polygons show U.S. Census Block Groups by three categories of *E. coli* percent susceptibility to two antibiotics. All included Block Groups have at least 30 isolates. ^b^Colored polygons show U.S. Census Block Groups that have statistically significant variation in antibiotic susceptibility. Blue cold spots show U.S. Census Block Groups with low susceptibility. Red hots spots show U.S. Census Block Groups with high antibiotic susceptibility. Hot spot analysis incorporates how each polygon relates to the mean antibiotic susceptibility and surrounding polygons (spatial dependencies). The color scale within the red hot-spots and blue cold-spots shows categories of statistical confidence. All included Block Groups have at least 30 isolates. Figures created with ArcGIS Pro software (Version 2.7; ESRI, Redlands, California, https://www.esri.com/en-us/arcgis/products/arcgis-pro/overview). *This geographic data presentation is considered ‘de-identified’ by ‘Expert determination’ under the HIPAA privacy rule.

**Figure 3:**
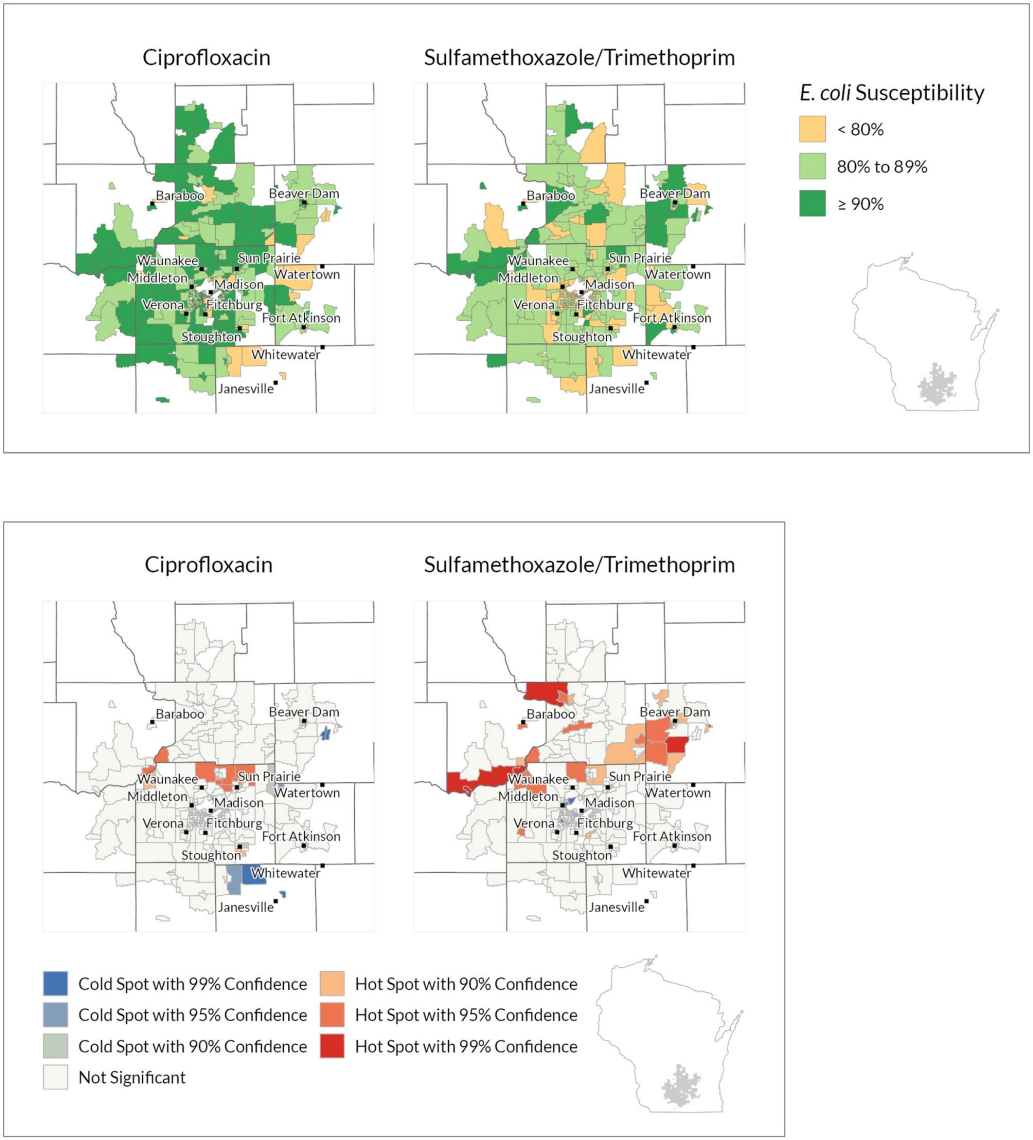
UW Health ciprofloxacin and sulfamethoxazole/trimethoprim susceptibility choropleth (noncontiguous areas included)^a^ and hot spot analysis results (Contiguous edge only)^b^*. ^a^Colored polygons show Block Groups by three categories of *E. coli* percent susceptibility to two antibiotics. All included Block Groups have at least 30 isolates. ^b^Colored polygons show Block Groups that have statistically significant variation in antibiotic susceptibility. Blue cold spots show Block Groups with low susceptibility. Red hots spots show Block Groups with high antibiotic susceptibility. Hot spot analysis incorporates how each polygon relates to the mean antibiotic susceptibility and surrounding polygons (spatial dependencies). The color scale within the red hot-spots and blue cold-spots shows categories of statistical confidence. All included Block Groups have at least 30 isolates included. Figures created with ArcGIS Pro software (Version 2.7; ESRI, Redlands, California, https://www.esri.com/en-us/arcgis/products/arcgis-pro/overview). *This geographic data presentation is considered ‘de-identified’ by ‘Expert determination’ under the HIPAA privacy rule.

**Figure 4:**
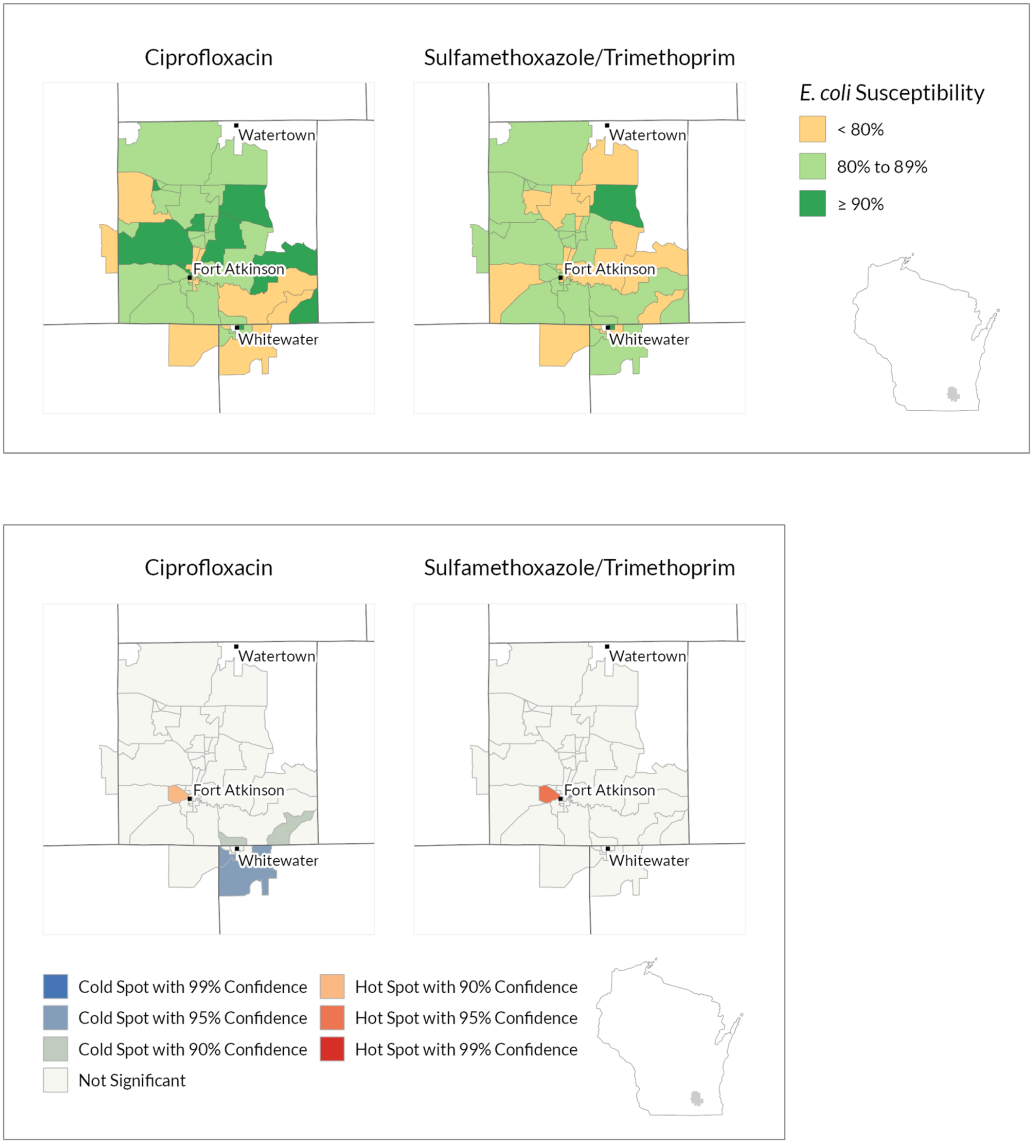
Fort healthcare ciprofloxacin and sulfamethoxazole/trimethoprim susceptibility choropleth (noncontiguous areas included)^a^ and hot spot analysis results (Contiguous edge only)^b^*. ^a^Colored polygons show Block Groups by three categories of *E. coli* percent susceptibility to two antibiotics. All included Block Groups have at least 30 isolates. ^b^Colored polygons show Block Groups that have statistically significant variation in antibiotic susceptibility. Blue cold spots show Block Groups with low susceptibility. Red hots spots show Block Groups with high antibiotic susceptibility. Hot spot analysis incorporates how each polygon relates to the mean antibiotic susceptibility and surrounding polygons (spatial dependencies). The color scale within the red hot-spots and blue cold-spots shows categories of statistical confidence. All included Block Groups have at least 30 isolates. Figures created with ArcGIS Pro software (Version 2.7; ESRI, Redlands, California, https://www.esri.com/en-us/arcgis/products/arcgis-pro/overview). *This geographic data presentation is considered ‘de-identified’ by ‘Expert determination’ under the HIPAA privacy rule.

The choropleth maps provided a data visualization of how susceptibility varied among the three clinically meaningful categories at the Block Group level (Figs. 2, 3, 4). This data visualization creates a unique “spatially-enabled” antibiogram, with data classification indicated by color for three categories (< 80, 80 to 90, ≥ 90%). These clinical thresholds are levels where a clinician may consider an alternative antibiotic as the acceptability of risk varies by context (e.g. severity of infection). The overall range of *E. coli* susceptibility to each antibiotic by Block Group varied by health system; the mean antibiotic susceptibility, susceptibility by Block Group range, and standard deviations are presented in the Table 1.

### Clustering

Antibiotic susceptibility at UW Health was spatially clustered. A positive spatially clustered pattern was identified from the UW Health data for both ciprofloxacin (Global Moran’s I = 0.096, p = 0.005) and TMP/SMX susceptibility (Moran’s I = 0.180, p < 0.001; see Supplemental Figs. S2, S3). The Global Moran’s I results for Fort HealthCare and MCHS were insignificant, indicating the distribution of antibiotic susceptibility was likely random for TMP/SMX and ciprofloxacin.

### Hot spot analysis

The hot spot analysis identifies which Block Groups are statistically significant for differences in antibiotic susceptibility considering the mean value and surrounding Block Groups. At the local level, we identified hot and cold spots with 90%, 95%, and 99% confidence at all three health systems (Figs. 2, 3, 4). Red hot spots (high susceptibility) and blue cold spots (low susceptibility) are identified in relation to the mean value and neighboring effects in spatial data, independent of the categories set for the choropleth susceptibility map. As such, hot and cold spots can occur within and outside of clinical threshold categories. For example, a hot spot at the highest confidence level signifies that the middle and its surrounding area are significantly greater than the mean, in some areas with 99% statistical confidence interval, independent of our chosen clinical thresholds of 80% and 90%. In this way, a block group with greater than 90% susceptibility, clinically high, could still be identified as a cold spot, with the cold spot block group and surrounding Block Groups trending below the mean.

The results at each health system varied between the two antibiotics. The MCHS results show a grouping of several ciprofloxacin hot spots in the southwest corner of the service area (Fig. 2). There are also hot spots scattered throughout rural Block Groups. Several adjacent Block Groups in northwestern Wisconsin are identified as ciprofloxacin cold spots and also correlate to lower antibiotic susceptibility on the choropleth map. From the UW-Health SMX/TMP data (Fig. 3), several larger more rural Block Groups north of Madison’s city center were identified as hot spots (red = high susceptibility). UW Health is based in Madison and Madison is in the middle of its service area. Cold spots (blue = lower susceptibility) were closer to the city center. Most of the ciprofloxacin hot and cold spots were identified in areas outside of Madison. While the Fort HealthCare choropleth map shows variation in susceptibility across all three categories, only a few Block Groups were identified as statistically significant hot or cold spots, consistent with the smaller sample size and service area (Fig. 4, Table 1).

## Discussion

This study provides spatially enabled antibiogram results and novel methods that can similarly be applied at a local level in other regions. Comparing the hot spot analysis and choropleth maps side by side—there appears to be more variation that is clinically meaningful than statistically significant. Block Groups that are both at the extreme end of the clinical range (below 80% susceptibility) and significantly below the mean (blue cold spots) are likely to be of greatest public health and clinical importance. However, their clinical relevance is yet to be determined. We compiled multiple years (2009–2018) of data to create a robust dataset to develop the method described here for creating a Block Group antibiogram. The results are not intended to immediately impact clinical care, but nevertheless provide preliminary data on the feasibility of this approach. The results support data collection from these and additional health systems to build localized antibiograms with more timely data. Aggregating data from multiple health systems within a Census Block group could create sufficient sample sizes for more timely aggregations, such as the past year, consistent with traditional annual antibiograms.

Our study has some limitations. First, the results are limited by the number of participating health systems and their geographic service area. The results are limited to data from patients with access to these health systems. Regions outside the service area of the participating health systems are missing, such as more rural south-western Wisconsin and the urban greater Milwaukee area in south-eastern Wisconsin. The geographic area included may not have enough variation to detect more spatial variability. Second, data for the three health systems was analyzed separately for this study, and it is plausible that some patients may visit more than one of the included health systems. If data from multiple health systems is combined during future projects, duplicate samples from the same individuals across health systems should be identified and managed, if feasible. Finally, one trade-off to using the small Block Groups compared to Census Tracts or counties is that it limits the number of isolates included in the study compared to the Census Tract level. However, using the Block Groups allows for more precise geographical detection of statistical differences in antibiotic susceptibility.

Antimicrobial resistance requires a coordinated response from across sectors locally and globally^22^. Thus, healthcare providers, policy makers and the communities served may take interest in our results and methods. Wisconsin is known for its agriculture and dairy industry, where antibiotics were once used in animal husbandry for growth-promotion, in addition to treat and prevent infections^23–26^. However, data on the degree of AMR transmission, especially among humans, animals, and the environment remains limited^26^. From this study, we cannot explain why the hot and cold spots exist, but future research will examine potential contributing factors, such as rurality and demographics, perhaps associated with higher and lower antibiotic susceptibility at U.S. Census Block Group levels. Ultimately, with a consideration of underlaying data limitations, geographic differences could be incorporated into clinical decision support tools for making empiric treatment decisions as we demonstrated in our previously published prototype design^12^.

The statistically significant hot and cold spots identified in this study are likely related to the complex intersection of health and demographics, such as healthcare utilization, antibiotic use, and environment. Further research is needed on whether and how apparent differences could influence empiric antibiotic treatment decisions.

## Methods

Three regionally-distinct Wisconsin health systems participated in the study. UW Health and Fort HealthCare serve south-central Wisconsin and the Marshfield Clinic Health System (MCHS) serves north-central Wisconsin. The University of Wisconsin-Madison Health Sciences Institutional Review Board (IRB, ID 2018-1305) and the Marshfield Clinic Research Institute IRB approved this study. All methods were carried out in accordance with relevant guidelines and regulations. The University of Wisconsin-Madison Health Sciences Institutional Review Board IRB determined this study qualified for a waiver of informed consent. This study used retrospective data that exists in the electronic health record and microbiology reports. The study posed minimal risk to subjects because the activities were limited to use of data from medical records and there were sufficient measures in place to protect the data, including expert determination of de-identification.

### Data collection

Data variables collected from each health system included antibiotic susceptibility results, details about the laboratory sample (pathogen, site, source, date), patient age, and patient address. Complete microbiology results were requested directly from the microbiology department as the medical records may not show all antibiotic results. Sometimes broad-spectrum antibiotics are suppressed. We also found that exporting microbiology results from the Electronic Health Record (Epic Systems) did not produce a line-by-line organized output file that could be easily imported for further data processing. Thus, data extraction at the health systems was more complex and time-intensive than expected with two data pulls/requests: (1) microbiology results for adult patients (18–89 years old) within the period of data collection, 2009–2018, and (2) medical records data (patient address, age). These two data requests were joined on the health system side and then unnecessary patient identifiers were removed before data transfer to ensure data anonymity. The 2009–2018 timeframe was selected to address the challenge of having a sufficient sample size for our analysis.

We included the initial *E. coli* isolate per patient for each unique year and source included with a patient address in Wisconsin. A study team member at the Marshfield Clinic Health System (coauthor BO) identified the initial unique isolates and excluded isolate duplicates prior to data transfer. Coauthor LL identified the initial unique isolates and excluded duplicates for the UW Health and Fort HealthCare datasets after data transfer utilizing unique address and age as a proxy for individual identifier (names and MRNs not collected).

We focused on *E. coli* for this study as nearly half of all isolates with antibiotic susceptibility results are *E. coli*. We examine *E. coli* susceptibility to two antibiotics, ciprofloxacin and sulfamethoxazole/trimethoprim (TMP/SMX), because of the range of susceptibility in Wisconsin and potential relevance to clinical practice. These antibiotics are well-known and utilized in both in-patient and ambulatory care settings.

### Data processing

Each health system dataset was processed separately and prepared for separate analysis. We did not evaluate if patients had laboratory samples taken at more than one of the included health systems.

Unique study identifiers were added to the transferred dataset. Patient addresses and unique study identifiers were separated from the clinical data. Patient addresses were geocoded to coordinates systematically with ArcGIS Pro software (Version 2.7; ESRI, Redlands, California) and a geocoding service made available to the study team by the Wisconsin Legislative Technology Services Bureau. This geocoding tool was selected because it is regularly updated, and the scale of the geocoding fit our project with data primarily from across Wisconsin. Full addresses were input into the geocoding tools including street number, street pre-directional (N, S, E, W), street name, street type, city, state, zip code, and zip + 4 codes. Addresses outside of Wisconsin were excluded. Point coordinates were then merged with laboratory data by the unique project identifier code.

We use Block Groups as the geographic unit for this study, which generally contain between 600 and 3000 people^27^. Block Groups are clustered together within the outer boundary of each U.S. Census Tract, and are the smallest geographical unit that provides population-related data in the United States. Point coordinates and shape files from the geocoding step were then spatially joined with Block Groups.

### Percent susceptibility calculation

Block Groups with a sample size of 30 or more *E. coli* isolates were included for further analysis; Block Groups with less than 30 isolates were excluded. We adopted the Clinical & Laboratory Standards Institute (CLSI) guideline standard of having at least 30 isolates per species for annual antibiograms to be the minimum number of isolates for a Block Group over the study period to be included in analysis (Section 7.2.2)^28^. This number is used “to obtain a reasonable statistical estimate of cumulative %S rates” (Section 6.4)^28^. Per CLSI consensus guidelines, it is acceptable to combine analysis over a longer period when there is less than 30 isolates encountered during a year^28,29^. In some areas, this step excluded entire census tracts that did not have any Block Groups that met this criterion.

Breakpoint criteria used for categorical interpretations (S, I, R) at each testing site were those of the CLSI that were in use at the time testing was performed. Interpretations were not retrospectively adjusted to account for updates in minimum inhibitory concentration breakpoints during the study period. Susceptibility results were assigned to binary inputs, where susceptible = 1, and resistant or intermediate = 0. Percent susceptibility for each antibiotic was then calculated by Block Group. For each health system, we also calculated mean, standard deviation, minimum susceptibility, and maximum susceptibility.

### Data visualization and analysis

The proportion of *E. coli* susceptible to each antibiotic, ciprofloxacin and TMP/SMX, by Block Group was visualized with a choropleth map for each health system. Clinician collaborators provided input on meaningful class breaks or thresholds for the map. We assigned three clinically meaningful data classification breaks for the color scale: less than 80%, 80 to 90%, and greater than 90% susceptibility. Choropleth maps were created with ArcGIS Pro Version 2.7.

We used a Global Moran’s I spatial autocorrelation method to evaluate the distribution of antibiotic susceptibility summarized by Block Groups. That is, are they evenly distributed, randomly distributed, or clustered^30^. The Global Moran’s I analysis quantifies how similar one object is to the surrounding objects (dispersed = − 1, random = 0, clustered =  + 1). We used the contiguous polygon approach when calculating Moran’s I with ArcGIS’ spatial autocorrelation tool.

We also computed the Getis-Ord Gi* index to identify local hot spots and cold spots, which are local clusters of Block Groups with statistically significant higher and lower susceptibilities^31,32^. We used contiguity edges for our conceptualization of spatial adjacency. A geographic example of hot spot analysis and interpretation is shown in Supplement Fig. S1. Hot spots with significantly higher (red) or lower (blue) percent susceptibility values are identified with two colors. Color gradations show the degree of statistical confidence (90%, 95%, 99%). For example, all red areas are statistically significant hot spots for higher percent susceptibility, and the shade of red indicates the level of statistical confidence.

The Strengthening the Reporting of Observational Studies in Epidemiology (STROBE) guidelines were reviewed as a checklist for describing our observational research^33^. Mirador Analytics, an external consulting firm, reviewed the geographic data presentation at the Block Group level and provided a data de-identification attestation expert report that stated the results can be considered ‘de-identified’ by ‘Expert Determination’ under the HIPAA Privacy Rule 45 CFR §164.514(b)^34^.

## Supplementary Information

Supplementary Figures.

## Data Availability

The datasets generated during and/or analyzed during the current study are not publicly available due to the terms of data collection with the health systems. However, de-identified data aggregates are available from the corresponding author on reasonable request.
