# Supplementary material for: A geospatial approach to identify patterns of antibiotic susceptibility at a neighborhood level in Wisconsin, United States
Source: Sci Rep. 2023 May 2;13:7122. doi: 10.1038/s41598-023-33895-5 (PMC10154319; doi:10.1038/s41598-023-33895-5)

Supplemental Material Document

Legenza, et. al. A Geospatial Approach to Identify Patterns of Antibiotic Susceptibility at a Neighborhood Level in Wisconsin, United States

Contents:

1. Supplement Figure 1 (**Figure S1**). Geographic example of hot spot analysis and interpretation.
2. Supplement Figure 2 (**Figure S2**). Results from Moran's Index analysis of UW Health data identifying geographically clustered ciprofloxacin susceptibility results.
3. Supplement Figure 3 (**Figure S3**). Results from Moran's Index analysis of UW Health data identifying geographically clustered sulfamethoxazole/trimethoprim susceptibility results.

Supplement Figure 1 (Figure S1). Geographic example of hot spot analysis and interpretation.

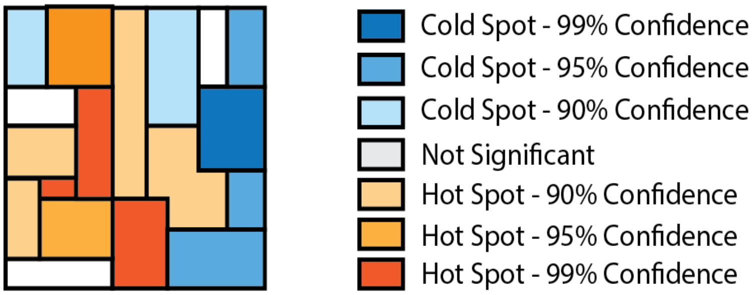

Supplement Figure 2 (Figure S2). Results from Moran's Index analysis of UW Health data identifying geographically clustered ciprofloxacin susceptibility results.

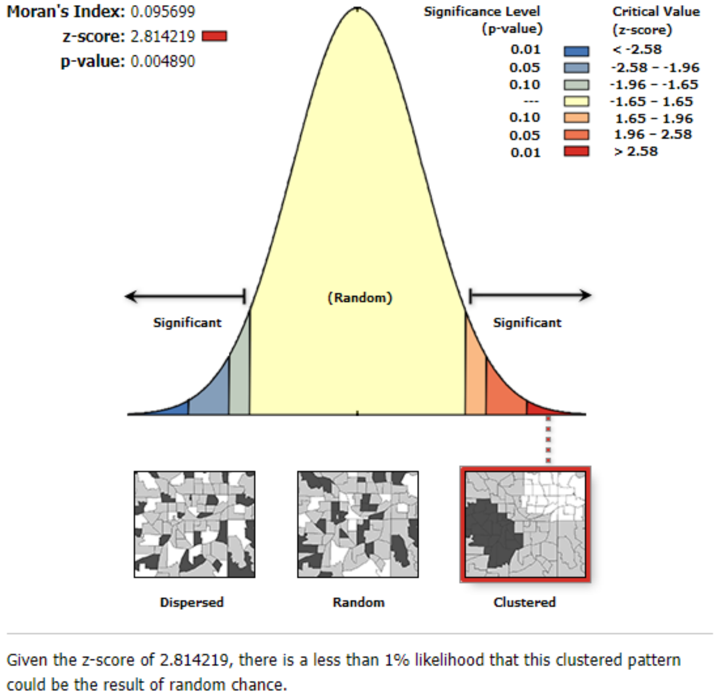

Supplement Figure 3 (Figure S3). Results from Moran's Index analysis of UW Health data identifying geographically clustered sulfamethoxazole/trimethoprim susceptibility results.

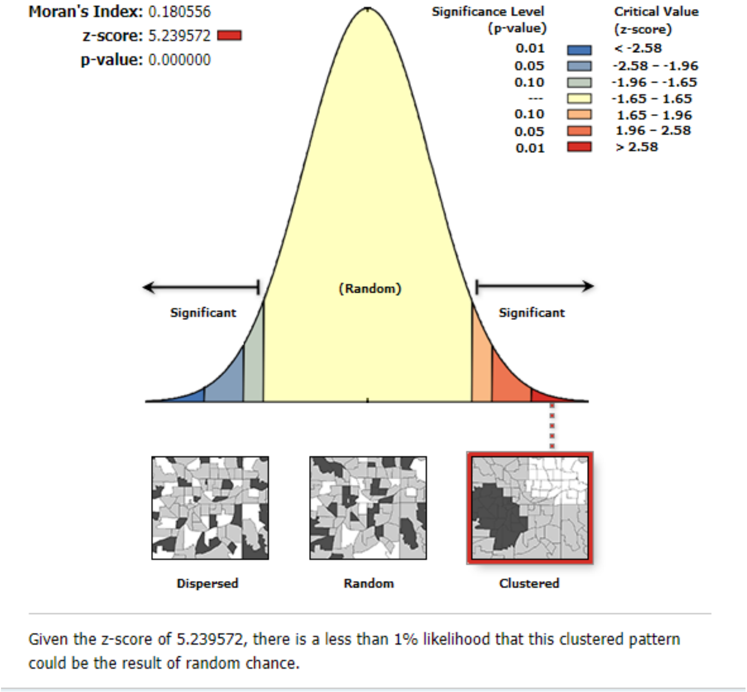

Supplement: Supplementary file 1 — Supplementary Figures. [file 41598_2023_33895_MOESM1_ESM.pdf]
